# Comparison and Analysis of Epidemiologic Characteristics of Stroke in Sichuan Province, China

**DOI:** 10.3389/fneur.2020.00877

**Published:** 2020-08-27

**Authors:** Qian Liu, Xinyin Xu, Jinghuan Fang, Ying Deng, Li He

**Affiliations:** ^1^Department of Neurology, West China Hospital, Sichuan University, Chengdu, China; ^2^Sichuan Center for Disease Control and Prevention, Sichuan, China

**Keywords:** stroke, prevalence, incidence, mortality, risk factors

## Abstract

Previous studies have shown that there is a geographic variation in the prevalence of stroke, with a lower prevalence of stroke in Sichuan province. And a stroke transition was found during the period of economic development as well. However, as the center of Southwest China, with a greatly developed economy, whether the geographic variation remained with lower burden of stroke in Sichuan province is unknown. Therefore, in this study, we analyzed the secular stroke status in Sichuan province to help explore the potential reasons for geographic disparity. From a cross-sectional study conducted based on eight national disease surveillance points (DSPs) in Sichuan province in 2013, the epidemiologic data of stroke were collected. Data of risk factors were obtained from a cross-sectional study based on 12 national DSPs in Sichuan province in 2013. The results showed that the age-standardized prevalence, incidence, and mortality of stroke in Sichuan province were 338.6/100,000 people [95% confidence interval (CI), 267.8–409.4], 147.1/100,000 person-years (95% CI = 100.6–193.6), and 72.4/100,000 person-years (95% CI = 40.0–104.8), respectively, which were significantly lower than those determined from the contemporary data of China in 2013. The analysis of the risk factors showed that the weights of contribution of the potential risk factors to stroke were in consistency with those published reports from other areas. In conclusion, the disparity of lower stroke burden in Sichuan than the average China remained, although with the great developments in Sichuan province over all those decades. In addition to traditional modifiable factors, we suggest that unknown or intrinsic differences such as genetic factors might play an important role in geographic disparity, which should be investigated in future studies.

## Introduction

Stroke is one of the leading causes of death and the major cause of adult disability worldwide, bringing a huge burden to our society. According to a recent study, the mean global lifetime risk for stroke from the age of 25 years onward increased from 22.8% in 1990 to 24.9% in 2016, and there was geographic variation, with the highest lifetime risk for stroke being 39.3% in China (1). Studies have also shown that the stroke burden in China has increased considerably over the past 30 years, and stroke was the leading cause of death in China and caused 1.7 million deaths in 2010 (2, 3). In addition, geographic variation exists not only in China but also worldwide (1, 2, 4). The reasons for the north-to-south and east-to-west gradients in the incidence of stroke in China are not clear at present, and the lowest incidence of stroke is in Southwest China (2, 5). To further explore the reasons for the geographic disparities and improve the effects of preventive measures, studies about epidemiological features, etiology, and prevention are of great importance.

Sichuan province is the center and the most developed province in Southwest China (including Sichuan, Yunnan, Guizhou, Tibet, and Chongqing province); data on stroke burden in Sichuan province have been shown in a limited number of studies conducted 20 years ago, and a lower morbidity and mortality of stroke were reported in this province than in other areas of China (6, 7). Many studies have indicated that the characteristics of the stroke transition were found during the period of economic development in China (2, 8, 9). In the past 20 years, Sichuan has experienced additional large socioeconomic context changes; there was an increase in the urbanization rate by more than 20%, and the rate reached up to 40.18% in 2010 (10). In addition, Sichuan's total gross domestic product (GDP) grew dramatically from 0.34 trillion yuan in 1998 to 2.64 trillion yuan in 2013 and to 3.70 trillion yuan in 2017 (11), and the GDP will continue increasing under the expansion of advanced commercial and tourism industries. In addition, Sichuan is famous for its special spicy dietary habits, as the residents' intakes of meats, oil, and fat are excessive (12). Considering these characteristics, is there still geographic disparity with a lower rate of stroke in Sichuan? No study has been conducted to answer this question. Hence, in this study, we compared the secular burden of stroke in Sichuan province with the entire country of China from 2012 to 2013 to help explore the potential reason for the geographic disparity.

## Materials and Methods

### Study Population and Design

**Figure 1:**
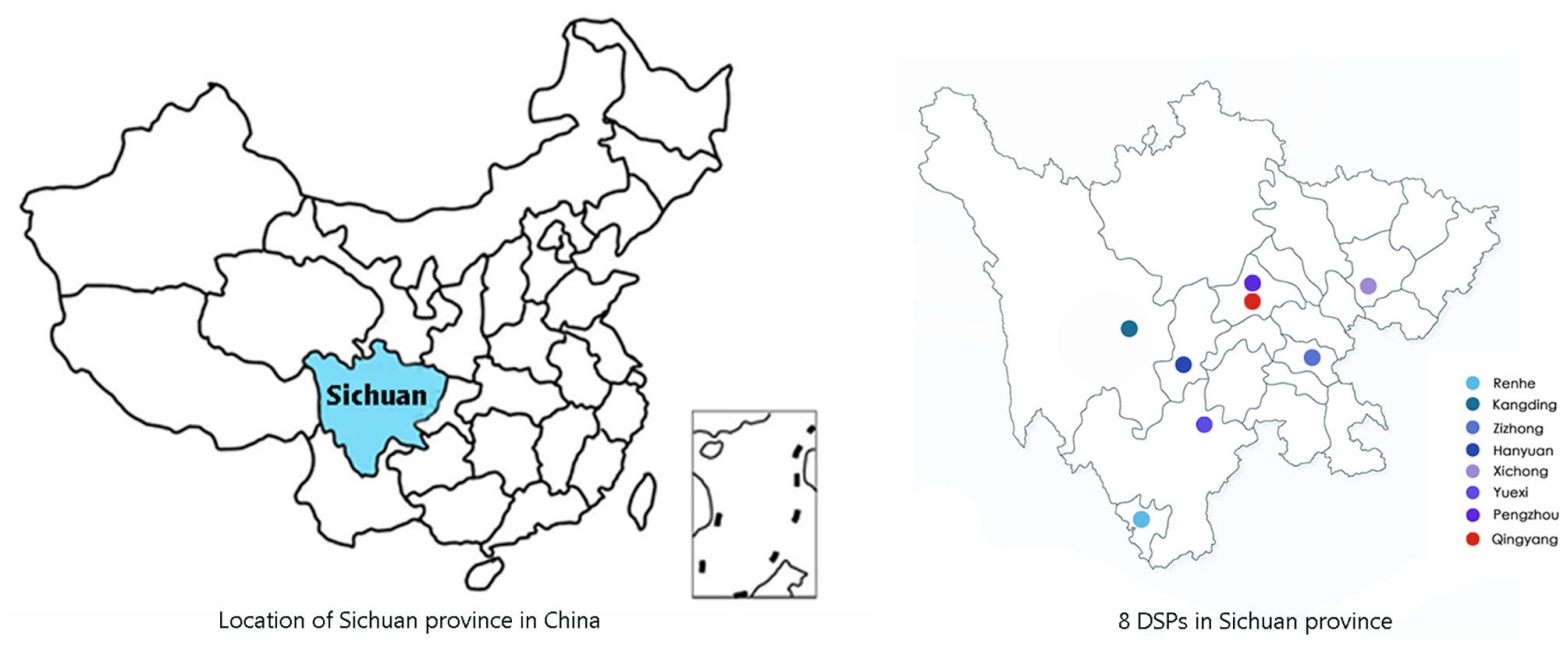
The location of Sichuan province in China and eight DSPs in Sichuan province.

To compare the morbidity and mortality of stroke between Sichuan province and the entire country of China, epidemiologic data on stroke were collected with a method similar to the national epidemiological survey on stroke (2). A detailed description of the methods, including the sample size calculation, sampling method, principles, and definitions, has previously been reported (2). A multistage, stratified clustering sampling technique was used to select individuals who had lived in the region for at least 6 months before the start of the survey. At the first stage of sampling, one district proportional to the population size of that area was selected in the survey sites. In the second stage, one or more urban communities/villages with a total population of more than 4,500 residents (at least 1,500 households) were selected in each selected district by using the random sampling method. Our study was conducted at eight monitoring sites (Figure 1) based on the national disease surveillance points (DSPs) system from September 1 to December 31 in 2013. In addition, at least 85% of these individuals were expected to complete the entire study protocol. Written informed consent was obtained from all study participants by the investigators.

There were 21,265 residents 20 years or older who were interviewed face-to-face by the Centers for Disease Control and Prevention investigators who were trained in a unified way and qualified to conduct the interview. In addition, 110 residents died between September 1, 2012, and August 31, 2013, from all causes, and the deaths were verified and recorded. By August 31, 2013, the point-prevalence day, all living subjects diagnosed with stroke were considered to have a prevalent stroke (point prevalence). First-ever stroke cases (either fatal or non-fatal) occurring between September 1, 2012, and August 31, 2013, were identified as incident strokes. The number of people who died of stroke from September 1, 2012, to August 31, 2013, was used to calculate the mortality of stroke. The definitions or calculations of prevalence, incidence, and mortality were consistent with the published national epidemiological study (2).

To help explore the possible causes of the stroke geographic disparity, we further analyzed and described the causal attribution of incidence of stroke to potential risk factors, the population attribution fractions (PAFs), in Sichuan province based on another cross-sectional study to identify the contributions of potential risk factors to stroke incidence at population level in Sichuan province. The cross-sectional stroke risk factors survey was conducted in 2013, which was a multistage, stratified cluster sampling study designed to investigate the level of modifiable cardiovascular risk factors in Sichuan province. The representative sample size was calculated considering economic status, population density, educational level, and crude mortality and covered 12 DSP (parts of 31 national DSPs, including the above eight monitoring sites for burden of stroke) in Sichuan province. More and detailed information can be found in a previously published article (13). Written informed consent was obtained from the participants.

### Ethics

The study about the epidemiology of stroke in Sichuan province was approved by the ethical review committees of Beijing Tiantan Hospital and West China Hospital of Sichuan University. The data collection protocol for the risk factors was approved by the ethics committee of the Chinese National Center for Chronic and Non-communicable Disease Control and Prevention.

### Diagnostic Criteria

As in the reported national epidemiologic study of stroke in China (2), stroke was defined as “rapidly developing clinical signs of focal (or global) disturbance of cerebral function, lasting more than 24 h or leading to death, with no apparent cause other than that of vascular origin,” which was based on the World Health Organization criteria (14). Then, the stroke cases were further classified into four major pathological types: subarachnoid hemorrhage, intracerebral hemorrhage, ischemic stroke, and an undetermined type. Stroke cases for which brain imaging was not performed after stroke onset and cases for which the results of the imaging or autopsy study were not available for review by the study neurologist were classified as stroke cases of an undetermined pathological type (2).

The PAFs of risk factors to stroke incidence were estimated using comparative risk assessment strategy with counterfactual analysis, in which a theoretical minimum risk exposure level (TMREL) was used as the counterfactual to estimate the burden of stroke associated with any exposure to potential risk factors that exceeded this level (15, 16). This approach is well-established and has been applied in global, national, and subnational level studies (15, 17, 18). Using this method, we compared population of interest with actual (factual) exposure distribution with corresponding theoretical optimum distribution (counterfactual) and estimated the proportional reduction in risk of stroke incidence. It should provide a more accurate estimate of the stroke incidence attributable to potential risk factors considering the relationships between risk factors and diseases are continuous rather than categorical. Thus, based on GBD 2013 study, the following mean [standard deviation *(SD)*] values of the optimal exposure distributions were adopted in our study (15): the 119 (6) mmHg for high systolic blood pressure, 5.4 (0.3) mmol/L for high fasting plasma glucose, 4.8 (0.9) mmol/L for high total cholesterol, 23.0 (1) kg/m^2^ for high body mass index, 200 (30) g/d of fruit consumption for diet low in fruits, 350 (30) g/d of vegetables consumption for diet low in vegetables, 8,000 metabolic equivalents of task-min for low physical activity, and any previous or current tobacco habits for smoking.

### Statistical Analysis

The age- and sex-specific prevalence (per 100,000 people), incidence, and mortality (per 100,000 person-years) were estimated. The age-standardized prevalence, incidence, and mortality of stroke were calculated by the direct method using the data from the 2010 China population census as the standard, and 95% confidence intervals (CIs) were calculated by using the Poisson distribution. The χ^2^ test was used to assess the significance of the differences in prevalence, incidence, and mortality between males and females, or urban and rural areas. The significant differences in the age-standardized prevalence, incidence, and mortality of stroke between the two sexes or Sichuan province and all of China were calculated by the *Z* test:

$$\begin{array}{l} \text{Z}=\frac{\left|{\text{ASR}}_{1}-{\text{ASR}}_{2}\right|}{\sqrt{{{\text{SE}}_{{\text{ASR}}_{\text{1}}}}^{2}+{{\text{SE}}_{{\text{ASR}}_{\text{2}}}}^{2}}}(\text{ASR},\text{ age - standardized rate};\text{ SE},\\ \text{ standard}\;\text{error}) \end{array}$$

We used estimates of size of the association between exposure to a risk factor and the risk of stroke incidence (rate ratios) and the prevalence of related risk factors in Sichuan province to calculate the attribution proportion of the incidence of stroke to the exposure to each risk factor in view of the TMREL (15). The PAF was calculated using the following equation:

$$\begin{array}{l} (\text{for continous variables}){\text{ PAF}}_{\text{joasct }}=\\ \frac{{\int^{\;}}_{x=1}^{\text{u}}{\text{RR}}_{\text{joas }}(x){\text{P}}_{\text{jasct}}(x)\text{dx}-{\text{RR}}_{\text{joas }}({\text{TMREL}}_{\text{jas}})}{{\int^{\;}}_{x=1}^{\text{u}}{\text{RR}}_{\text{joas }}(x){\text{P}}_{\text{jasct}}(x)\text{dx}}\\ (\text{for categorical variables) }{\text{PAF}}_{\text{joasct }}=\\ \frac{{\sum^{\;}}_{x=1}^{\text{u}}{\text{RR}}_{\text{joas }}(x){\text{P}}_{\text{jasct}}(x)-{\text{RR}}_{\text{joas }}({\text{TMREL}}_{\text{jas}})}{{\sum^{\;}}_{x=1}^{\text{u}}{\text{RR}}_{\text{joas }}(x){\text{P}}_{\text{jasct}}(x)} \end{array}$$

[RR_joasc_ (*x*) is the relative risk as a function of the exposure level *x* for risk factor *j*, cause *o*, age group *a*, sex *s*, and country *c*. *l* is the lowest level of exposure, and *u* is the highest level of exposure observed. *P*_jasct_ (x) is the distribution of exposure for risk *j* in age group *a*, sex *s*, country *c*, and year *t*. TMREL_jas_ is the TMREL for risk factor *j*, age group *a*, and sex *s*. For continuous variables, definite integral was used to calculate the cumulative probability that the d*x* is the cutting width of the exposure level *x*].

## Results

### Demographic Characteristics

The demographic characteristics of the interviewed participants are shown in Table 1. The mean age of the interviewed participants was 46.90 ± 16.73 years. The rural residents accounted for 52.6% of the participants. The proportion of participants who completed primary school or a lower level of education was 54.7%. In addition, 80% of the participants were married or had a partner. A total of 90.1% (82 of 91 cases) of the participants with prevalent stroke and 70.0% (28 of 40 cases) of those with incident stroke were hospitalized for stroke within 7 days of stroke onset.

**Table 1:** Characteristics of the interviewed participants (≥20 years) in 2013.

**Characteristics**	**Overall**	**Men**	**Women**
Participants, *n (%)*	21,265 (100)	10,536 (49.5)	10,729 (50.5)
Age, mean *(SD)*, years	46.90 (16.73)	46.42 (16.39)	46.95 (16.78)
**Residence**, ***n (%)***			
Urban	10,089 (47.4)	4,877 (46.3)	5,212 (48.6)
Rural	11,176 (52.6)	5,659 (53.7)	5,517 (51.4)
**Age groups**, ***n (%)***			
20–29	4,071 (19.1)	2,018 (19.2)	2,053 (19.1)
30–39	3,695 (17.4)	1,845 (17.5)	1,850 (17.2)
40–49	5,076 (23.9)	2,589 (24.6)	2,487 (23.2)
50–59	3,114 (14.6)	1,522 (14.4)	1,592 (14.8)
60–69	3,071 (14.4)	1,501 (14.2)	1,570 (14.6)
70–79	1,659 (7.8)	812 (7.7)	847 (7.9)
≥80	579 (2.7)	249 (2.4)	330 (3.1)
**Education**, ***n (%)***			
Primary school or lower	11,623 (54.7)	5,502 (52.2)	6,121 (57.1)
Middle school	8,266 (38.9)	4,342 (41.2)	3,924 (36.6)
College and higher	1,350 (6.3)	687 (6.4)	672 (6.3)
Missing	26 (0.1)	14 (0.1)	12 (0.1)
**Marital status**, ***n (%)***			
Married	17,060 (80.2)	8,389 (79.6)	8,671 (80.8)
Single	2,473 (11.6)	1,527 (14.5)	946 (8.8)
Widowed	1,666 (7.8)	591 (5.6)	1,075 (10.0)
Missing	66 (0.3)	29 (0.3)	37 (0.3)
**Occupation**, ***n (%)***			
Student	373 (1.8)	183 (1.7)	190 (1.8)
Worker	852 (4.0)	464 (4.4)	388 (3.6)
Farmer	17,012 (80.0)	8,334 (79.1)	8,678 (80.9)
Employee	679 (3.2)	381 (3.6)	298 (2.8)
Entrepreneur	774 (3.6)	402 (3.8)	372 (3.5)
Retired or unemployed	1,518 (7.1)	742 (7.0)	776 (7.2)
Missing	57 (0.3)	30 (0.3)	27 (0.3)

### Stroke Status in Sichuan Province in 2012 to 2013

Among the 21,265 face-to-face interviewed participants, the number of prevalent stroke cases was 91 (428.1 per 100,000 people), and the 2010 China population census standardized prevalence was 338.6 per 100,000 people (Table 2). There were no significant differences between sexes in the age-specific and age-standardized prevalence (all *p* > 0.05).

**Table 2:** Prevalence (with 95% CIs) of stroke per 100,000 adults in Sichuan by sex in 2013.

**Age group (years)**	**Men**			**Women**				**Total**		
	**No. of strokes**	**Prevalence**	**95% CI**	**No. of strokes**	**Prevalence**	**95% CI**	***p***	**No. of strokes**	**Prevalence**	**95% CI**
20–29	0	0	(0–0)	0	0	(0–0)	—	0	0	(0–0)
30–39	0	0	(0–0)	0	0	(0–0)	—	0	0	(0–0)
40–49	4	154.5	(3.2–305.8)	3	120.6	(0–257.0)	1.000	7	137.9	(35.8–240.0)
50–59	8	525.6	(162.3–888.9)	8	502.5	(155.2–849.8)	0.928	16	513.8	(262.7–764.9)
60–69	18	1,199.2	(648.5–1,749.9)	15	955.4	(474.2–1,436.6)	0.512	33	1,074.6	(709.9–1,439.3)
70–79	17	2,093.6	(1,108.8–3,078.4)	11	1,298.7	(536.2–2,061.2)	0.209	28	1,687.8	(1,067.9–2,307.7)
≥80	3	1,204.8	(0–2,559.9)	4	1,212.1	(31.5–2,392.8)	1.000	7	1,209	(318.8–2,099.2)
Total	50	474.6	(343.4–605.8)	41	382.1	(265.4–498.8)	0.302	91	428.1	(340.4–515.9)
ASR^*^		372.3	(267.1–477.5)		305.0	(210.0–400.0)	>0.05		338.6	(267.8–409.4)

* *ASR, age-standardized rates based on the 2010 China population census*.

There were 40 incident stroke cases. The crude incidence was 187.1 per 100,000 person-years, and the 2010 Chinese population census standardized incidence was 147.1 per 100,000 person-years (Table 3). There were no significant differences between men and women in either the age-specific or the age-standardized incidence (all *p* > 0.05).

**Table 3:** Incidence (with 95% CIs) of stroke per 100,000 person-years among Sichuan adults by sex in 2012 to 2013.

**Age group (years)**	**Men**			**Women**				**Total**		
	**No. of strokes**	**Rate**	**95% CI**	**No. of strokes**	**Rate**	**95% CI**	***p***	**No. of strokes**	**Rate**	**95% CI**
20–29	0	0.0	(0–0)	0	0	(0–0)	—	0	0	(0–0)
30–39	1	54.0	(0–105.8)	0	0	(0–0)	1.000	1	27.0	(0–79.9)
40–49	1	38.6	(0–75.6)	1	40.2	(0–118.9)	1.000	2	39.4	(0–94.0)
50–59	2	130.9	(0–312.2)	4	250.9	(5.3–496.5)	0.688	6	192.2	(38.6–345.8)
60–69	8	526.7	(162.7–890.7)	4	253.0	(5.4–500.6)	0.220	12	387.1	(168.5–605.7)
70–79	6	729.9	(148.0–1,311.8)	6	697.7	(141.1–1,254.0)	0.937	12	713.4	(311.2–1,115.6)
≥80	3	1,123.6	(0–2,387.9)	4	1,159.5	(29.8–2,289.2)	1.000	7	1,143.8	(301.3–1,986.3)
Total	21	198.1	(113.4–282.8)	19	176.3	(97.1–255.5)	0.712	40	187.1	(129.2–245.0)
ASR^*^		152.4	(85.7–219.1)		142.1	(114.4–169.8)	>0.05		147.1	(100.6–193.6)

* *ASR, age-standardized rates based on the 2010 China population census*.

The number of deaths from stroke was 20, which accounted for 18.1% of all deaths. The crude mortality of stroke was 93.6 per 100,000 person-years, and the 2010 Chinese population census standardized mortality was 72.4 per 100,000 person-years (Table 4). There were no significant differences between sexes in either the age-specific or the age-standardized mortality (all *p* > 0.05).

**Table 4:** Mortality (with 95% CIs) of stroke per 100,000 person-years among Sichuan adults by sex in 2012 to 2013.

**Age group (years)**	**Men**			**Women**				**Total**		
	**No. of strokes**	**Rate**	**95% CI**	**No. of strokes**	**Rate**	**95% CI**	***p***	**No. of strokes**	**Rate**	**95% CI**
20–29	0	0.0	(0–0)	0	0.0	(0–0)	—	0	0.0	(0–0)
30–39	1	54.0	(0–159.8)	0	0.0	(0–0)	1.000	1	27.0	(0–79.9)
40–49	0	0	(0–0)	0	0.0	(0–0)	—	0	0	(0–0)
50–59	1	65.4	(0–193.6)	1	62.7	(0–185.6)	1.000	2	64.1	(0–152.9)
60–69	3	197.5	(0–420.8)	1	63.3	(0–187.3)	0.365	4	129.0	(2.7–255.4)
70–79	1	121.7	(0–360.0)	4	465.1	(10.4–919.8)	0.375	5	297.3	(37.1–557.5)
≥80	5	1,872.7	(246.7–3,498.7)	3	869.6	(0–1,849.3)	0.305	8	1,307.2	(407.3–2,207.1)
Total	11	103.8	(42.5–165.1)	9	83.5	(29.0–138.0)	0.628	20	93.6	(52.6–134.6)
ASR^*^		80.4	(31.5–129.3)		64.3	(21.9–106.7)	>0.05		72.4	(40.0–104.8)

* *ASR, age-standardized rates based on the 2010 China population census*.

**Figure 2:**
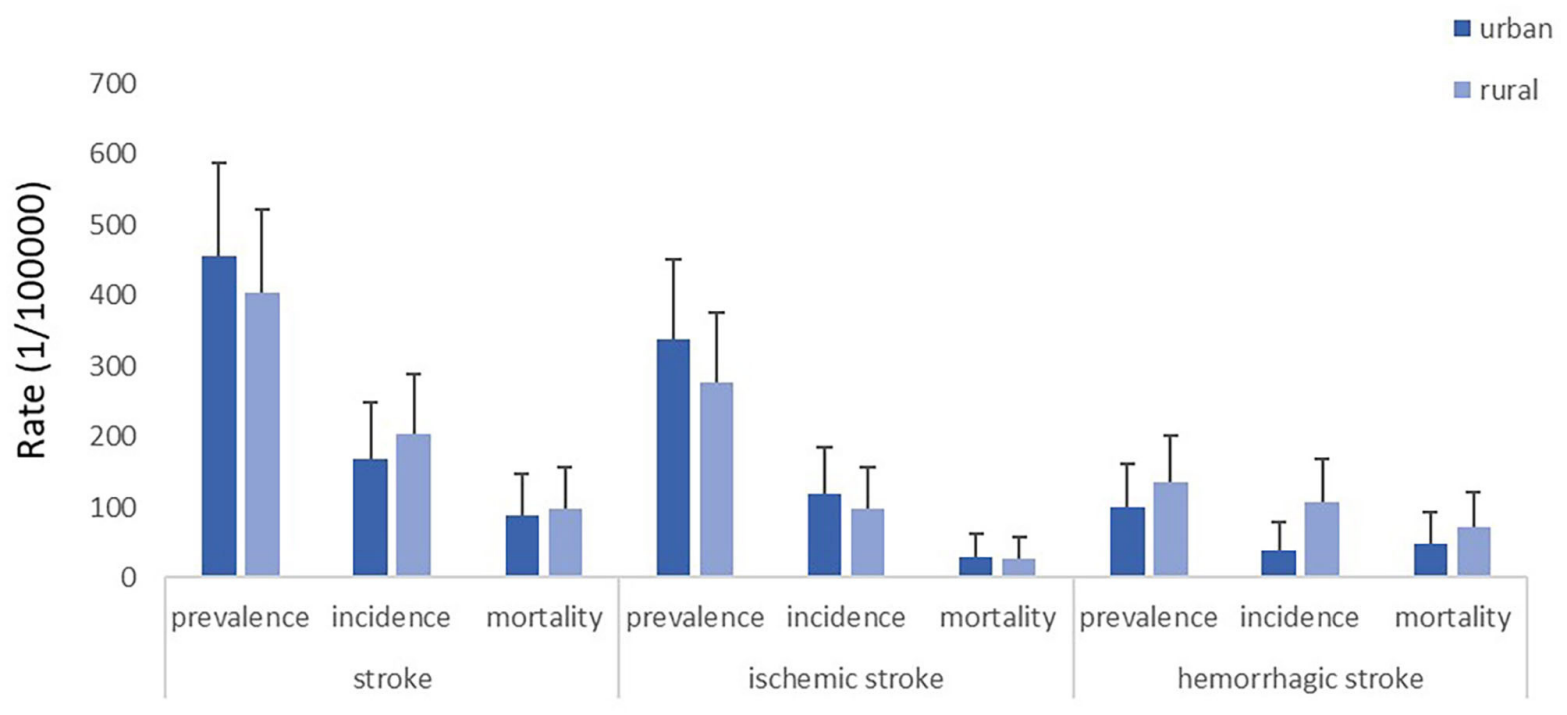
Crude prevalence, incidence and mortality of stroke, ischemic stroke, and hemorrhagic stroke in urban and rural areas of Sichuan province, respectively.

Besides, the crude prevalence, incidence, and mortality of stroke in urban areas were 455.94, 167.69, and 88.87 per 100,000 people, respectively, and in rural areas were 402.65, 204.68, and 97.89 per 100,000 people, respectively. We did not find significant differences in prevalence, incidence, and mortality of stroke and two stroke subtypes between urban areas and rural areas, although the burden of hemorrhagic stroke in rural areas was slightly higher than in urban areas (Figure 2).

### Stroke Subtypes

Among the prevalent and incident stroke cases, ischemic stroke was the most common subtype of stroke, with a proportion of 69.9% in prevalent stroke cases and 57.0% in incident stroke cases, followed by intracerebral hemorrhage, which had proportions of 26.9% and 35.0% in prevalent cases and incident cases, respectively. However, intracerebral hemorrhage was the major cause of death and accounted for 60% of deaths from stroke (Table S1).

### Stroke Burden in Sichuan Province Compared With Southwest China and Entire China

There was a gradient in the age-standardized prevalence, incidence, and mortality of stroke in Sichuan, Southwest China, and the entire China. The age-standardized prevalence, incidence, and mortality of stroke in Sichuan were lower than those in Southwest China, and those in Southwest China were lower than China (Figure S1). An analysis of the significance of these differences showed that there were significant differences between Sichuan and entire China in the age-standardized prevalence, incidence, and mortality of stroke (all *p* < 0.05, Figure S1).

### Risk Factors Analysis

The levels of the risk factors of the three age groups (18–44, 45–59, and >60 years old) in males and females in Sichuan province in 2013 are shown in Table 5. Among the eight risk factors (high systolic blood pressure, diet low in fruits, smoking, high body mass index, high total cholesterol, low physical activity, high fasting plasma glucose, diet low in vegetables) for ischemic stroke and six risk factors (high systolic blood pressure, diet low in fruits, smoking, high body mass index, high fasting plasma glucose, diet low in vegetables) for hemorrhagic stroke, high systolic blood pressure was the largest contributor to both ischemic and hemorrhagic stroke (Figures S2, S3). For ischemic stroke, smoking was the second most important factor, followed by blood pressure; high body mass index and high fasting plasma glucose level (<15%) were not the major contributors to the incidence of ischemic stroke, whereas the high total cholesterol level had the least importance (Figure S2). Additionally, for hemorrhagic stroke, a diet low in fruits was the second highest contributing factor, with contribution up to 40%, which was comparable to the contribution of smoking in males (Figure S3). There was a decline in the contribution of various risk factors to stroke with advancing age, and for individuals 80 years or older, the risk factors had little contributions to the incidence of stroke (Figures S2, S3).

**Table 5:** The level of related risk factors in Sichuan province in 2013.

**Factors (mean ± SD or %)**	**Male**			**Female**		
	**Age 18–44**	**Age 45–59**	**Age ≧60**	**Age 18–44**	**Age 45–59**	**Age ≧60**
Body mass index (kg/m^2^)	23.82 ± 3.46	23.95 ± 3.22	23.17 ± 3.15	23.53 ± 3.67	24.82 ± 3.51	23.91 ± 3.58
Total cholesterol (mmol/L)	4.81 ± 1.04	4.96 ± 0.95	4.93 ± 0.99	4.59 ± 0.94	5.05 ± 1.00	5.2 ± 0.98
Fasting plasma glucose (mmol/L)	5.61 ± 1.56	5.81 ± 1.65	5.84 ± 1.67	5.44 ± 1.24	5.79 ± 1.74	6.03 ± 1.75
Systolic blood pressure (mmHg)	124.56 ± 15.45	134.26 ± 19.88	142.15 ± 21.36	119.63 ± 17.27	132.83 ± 19.74	143.63 ± 22.90
Consumption of fruits (g)	109.37 ± 157.07	83.19 ± 148.29	63.67 ± 118.89	123.41 ± 151.18	90.68 ± 147.91	77.77 ± 136.54
Consumption of vegetables (g)	413.38 ± 296.71	395.39 ± 275.96	407.39 ± 281.91	388.84 ± 284.30	414.67 ± 301.93	401.53 ± 287.49
Smoking (%)	45.57	64.84	48.64	1.41	3.47	5.53
Low physical activity (%)	68.09	62.84	74.9	78.47	67.95	81.03

And the contributions of those risk factors for ischemic stroke were similar between rural and urban areas (Figure S4), whereas PAFs of smoking, high body mass index, and diet low in fruit and vegetables for hemorrhagic stroke seem to be slightly higher in rural areas than urban areas (Figure S5).

## Discussion

This is the first epidemiologic investigation on stroke in Sichuan in the last 20 years (6, 7). Our findings showed that the disparity remained, even with the great development of Sichuan province throughout these past decades. The age-standardized prevalence, incidence, and mortality of stroke in 2013 in Sichuan province were 338.6/100,000 people (95% CI = 267.8–409.4), 147.1/100,000 person-years (95% CI = 100.6–193.6), and 72.4/100,000 person-years (95% CI = 40.0–104.8), respectively, which were significantly lower than those according to contemporary data from China. And not like the higher stroke burden in rural than urban areas in China, there was no significant difference between rural and urban areas in Sichuan province. The weights of the contribution of these modifiable risk factors to stroke incidence in this study were similar to those presented in previous reports.

The age-standardized prevalence, incidence, and mortality of stroke were 1,115/100,000 people (95% CI = 997–1,233), 247/100,000 person-years (95% CI = 211–283), and 114.8/100,000 person-years (95% CI = 96.3–133.3) in China (2). These data indicated that there is still geographic disparity, with a significantly lower burden of stroke in Sichuan than in entire China. However, as the prevalence of stroke in China was 3.3-fold that in Sichuan and the incidence and mortality in China was approximately only 1.6-fold those in Sichuan, we speculate that there was a large increase in the incidence of stroke in Sichuan with the rapid development and urbanization of Sichuan province in those decades and that the small number of stroke survivors at baseline in Sichuan combined with the rapid increase in the incidence of stroke caused a larger gap in the prevalence than in the incidence between Sichuan and China. Furthermore, the incidence and mortality in 2013 were higher than those reported in a study conducted in 1986, which showed that the annual age-standardized incidence in Sichuan by 1960 US total population was 85.3/100,000 persons, and the mortality was 68.15/100,000 persons (7). A continuous monitoring study of stroke in Deyang city in Sichuan province also showed a fluctuating increase in stroke incidence, from 49.87/100,000 person-years in 1990 to 127.31/100,000 person-years in 2000 (19). In addition, the mortality of stroke in our study was in line with the study monitoring the deaths in Sichuan province that showed a standardized mortality of 94.51/100,000 person-years in 2011 and an increasing trend thereafter (20). Therefore, although with epidemiologic character of relatively lower stroke burden in Sichuan province at present, we should note that there is still an increase in the incidence and mortality of stroke with economic development, which also signifies an urgent need to prevent future stroke occurrences.

Similar to previous studies (3, 15), this study showed that the key contributors to stroke were high blood pressure, diet low in fruits, high body mass index, and smoking. Studies have shown that hypertension is the most important reason for geographic variability in stroke incidence and mortality in China (4, 7, 21). However, in the article that investigated the geographic distribution of stroke and hypertension, it should be noticed there was a lower incidence of stroke in Sichuan province but with a prevalence of hypertension as high as central China (7). In addition, studies have indicated that the prevalence of hypertension in Sichuan province was high, especially in rural areas (22, 23). Therefore, despite the consistency with previous studies that showed hypertension is the most important risk factor for stroke in China, with the population-attributable risk up to 53.2% (2, 4, 7, 24–26), we suggest that there might be some unknown protective factors contributing to a lower stroke burden in Sichuan province. Smoking, an independent risk factor for stroke (7, 27), was also the second highest contributing factor to the incidence of ischemic stroke in this study. Because there was a relatively higher prevalence of smoking (33.7%) in Sichuan province than at the average national level (7, 28), this result suggests that unknown protective factors contribute to a lower stroke burden in this area. Moreover, because of the dietary habits in Sichuan consisting of foods with excessive fat and calories (12), the body mass index (mean ≈24 kg/m^2^) and the total cholesterol (mean ≈5 mmol/L, higher than the average 4.5 mmol/L in China) were not low in Sichuan population, but these factors had a relative low contribution to the incidence of stroke in this population. In terms of the high prevalence of low physical activity, the contribution of which was even higher than that of a high body mass index, further supporting the idea that there are unknown protective factors for ischemic stroke in this population. The contribution of traditional risk factors to stroke was reduced with advancing age, as reported in several studies (29, 30), which suggests that there are some internal factors that make a certain group of people less vulnerable to suffering a stroke. But for people who are susceptible to stroke, the risk factors will inevitably impact them and eventually leading to a stroke when they reach 40–79 years old. However, we failed to collect data on genetic factors such as the family history, which were indicated in many studies to play an important role in the incidence of stroke (31, 32). A study conducted in West China in 2015 showed that among middle-aged and older farmers (≥40 years), family history was the strongest risk factor for stroke compared with hypertension and obesity (32). Moreover, a study of the differences in risk allele frequencies among 52 populations and geographic areas showed a significant geographic variation in eight single-nucleotide polymorphisms with the prevalence of cardiovascular disease (33). From the risk factor analysis, we speculate that there were other unknown protective factors, such as genetic differences, which caused a lower stroke burden in Sichuan province (34).

There are several potential limitations in this study. First, as a cross-sectional study, recall bias may affect the evaluation of the prevalence, incidence, and mortality of stroke. However, great efforts have been made to minimize the possibility for bias; the data obtained from the door-to-door interviews were cross-checked, people suspected of having stroke were re-interviewed carefully by trained neurologists, and brain imaging tests were arranged when required. Second, we did not record risk factors for stroke among all the participants when conducted the epidemiologic study of stroke, so we had to obtain representative risk factor data from another contemporary study. Additionally, we did not record all the risk factors (such as the family history), so the data were insufficient for us to identify the reason for geographic disparities regarding stroke.

## Conclusion

In our study, there was still geographic disparity in the prevalence, incidence, and mortality of stroke, which were significantly lower in Sichuan than in entire China, irrespective of rapid socioeconomic developments in Sichuan province over the past two decades. In addition to hypertension, we suggest that other unknown modifiable factors or intrinsic factors, such as genetic factors, might play an important role in the geographic disparity and the relatively lower stroke burden in Sichuan. Future studies on stroke in different areas are necessary to explore special protective or risk factors.

## Data Availability Statement

All datasets generated and analyzed for this study are included in the article/Supplementary Material.

## Ethics Statement

The studies involving human participants were reviewed and approved by ethical review committees of Beijing Tiantan Hospital, ethical review committees of South West China Hospital, and Ethics Committee of the Chinese National Center for Chronic and Non-communicable Disease Control and Prevention (NCNCD). The patients/participants provided their written informed consent to participate in this study.

## Author Contributions

LH and QL were involved in the study design. YD, LH, XX, and JF were responsible for the data collection. QL and XX analyzed data and wrote the manuscript. YD and LH modified and revised the manuscript. All authors have read and approved the final version of the manuscript.

## Conflict of Interest

The authors declare that the research was conducted in the absence of any commercial or financial relationships that could be construed as a potential conflict of interest.
