# Supplementary material for: Comparison and Analysis of Epidemiologic Characteristics of Stroke in Sichuan Province, China
Source: Front Neurol. 2020 Aug 27;11:877. doi: 10.3389/fneur.2020.00877 (PMC7481474; doi:10.3389/fneur.2020.00877)
Supplement: Supplementary file 1 [file Data_Sheet_1.docx]

Table S1 The proportion of different types of stroke

| Stroke types | prevalent stroke | incident stroke | mortal stroke |
| --- | --- | --- | --- |
| SAH | 1(1.0%) | 2(5.0%) | 1(5.0%) |
| ICH | 25(26.9%) | 14(35.0%) | 12(60.0%) |
| IS | 65(69.9%) | 23(57.5%) | 6(30.0%) |
| UND | 2(2.2%) | 1(2.5%) | 1(5.0%) |

IS: ischemic stroke; ICH: intracerebral hemorrhage; SAH: subarachnoid hemorrhage;

UND: stroke of an undetermined pathological type.


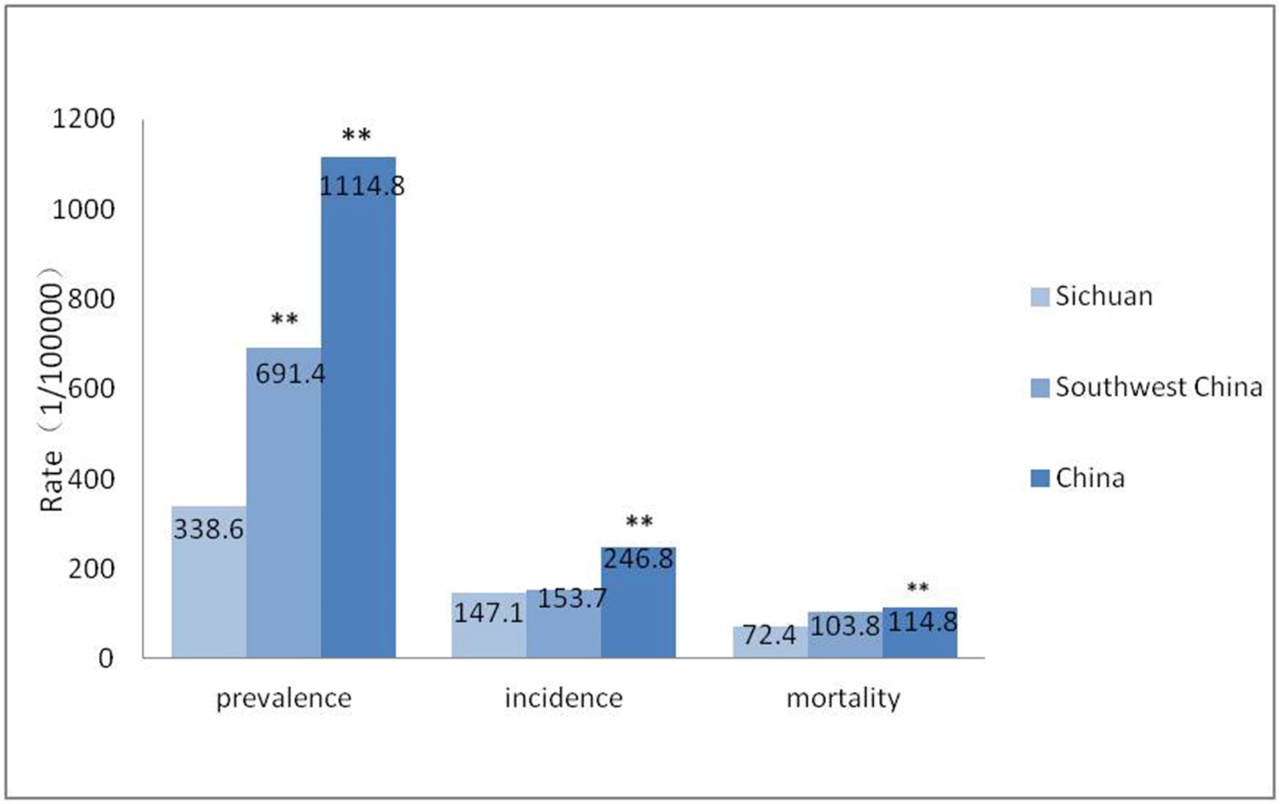


Figure S1 Age-standardized prevalence, incidence, and mortality of stroke in Sichuan compared to Southwest China and China in 2012 to 2013.

**: Compared with Sichuan p<0.05


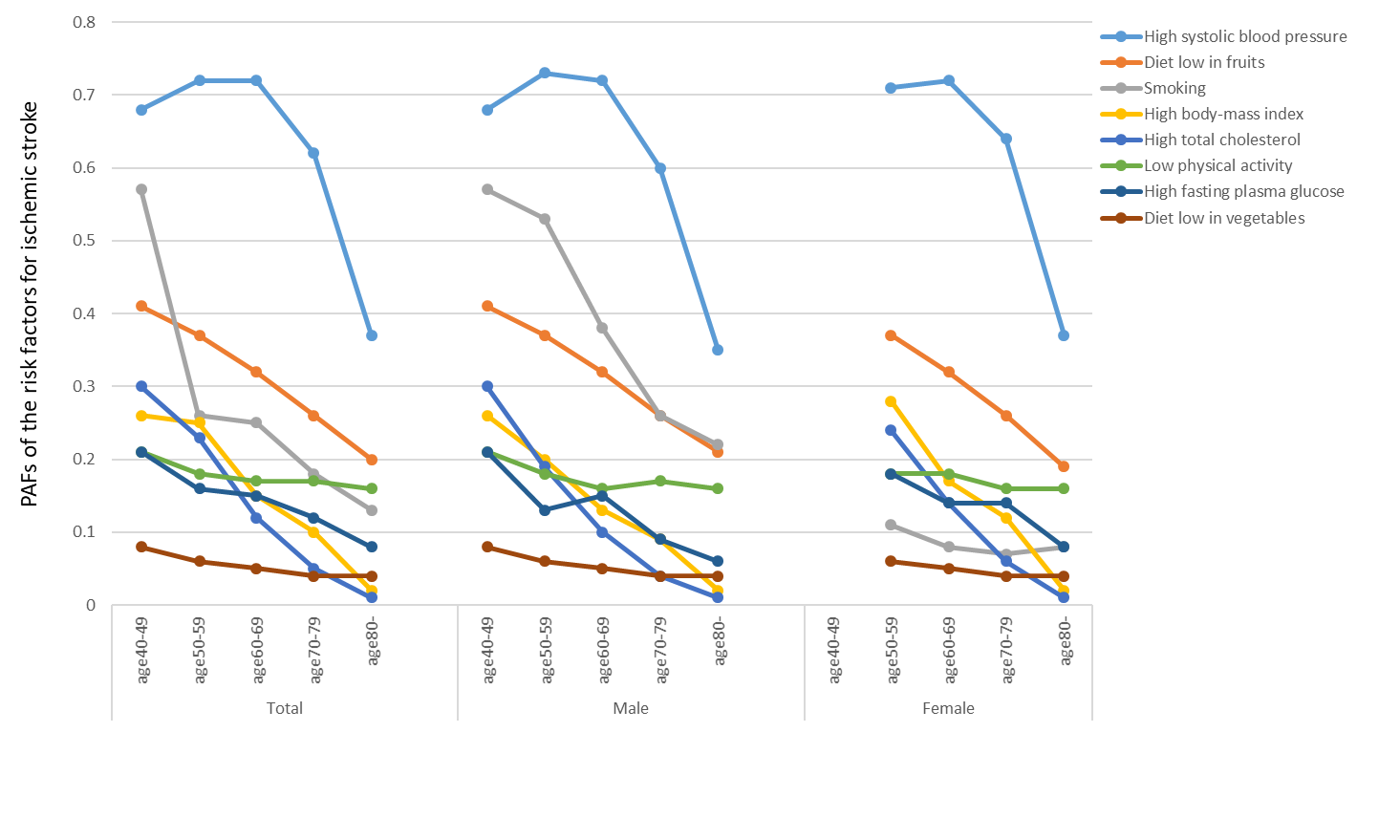


Figure S2. The PAFs of the risk factors for ischemic stroke.


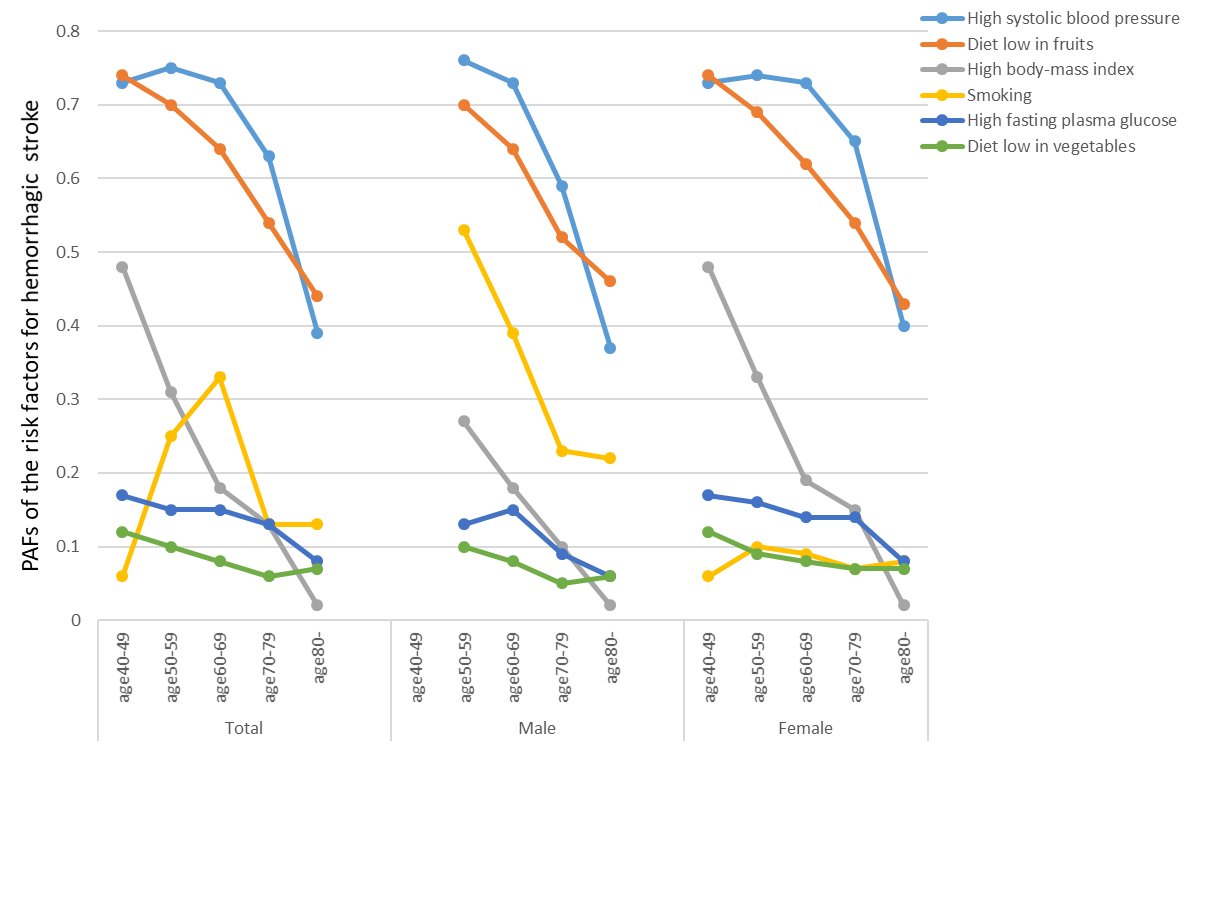
Figure S3. The PAFs of the risk factors for hemorrhagic stroke.


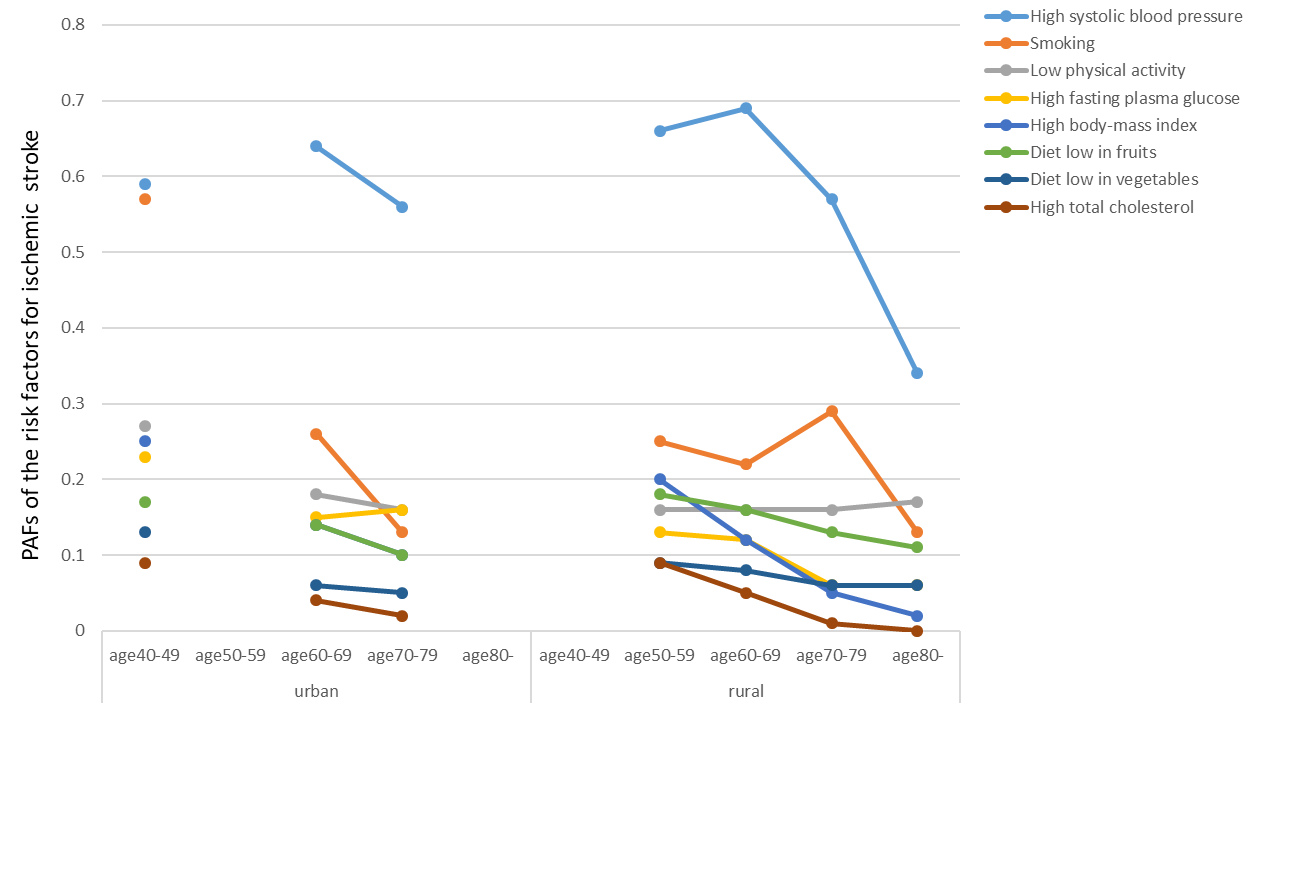


Figure S4. The PAFs of the risk factors for ischemic stroke in urban and rural areas of Sichuan province in 2012 to 2013.


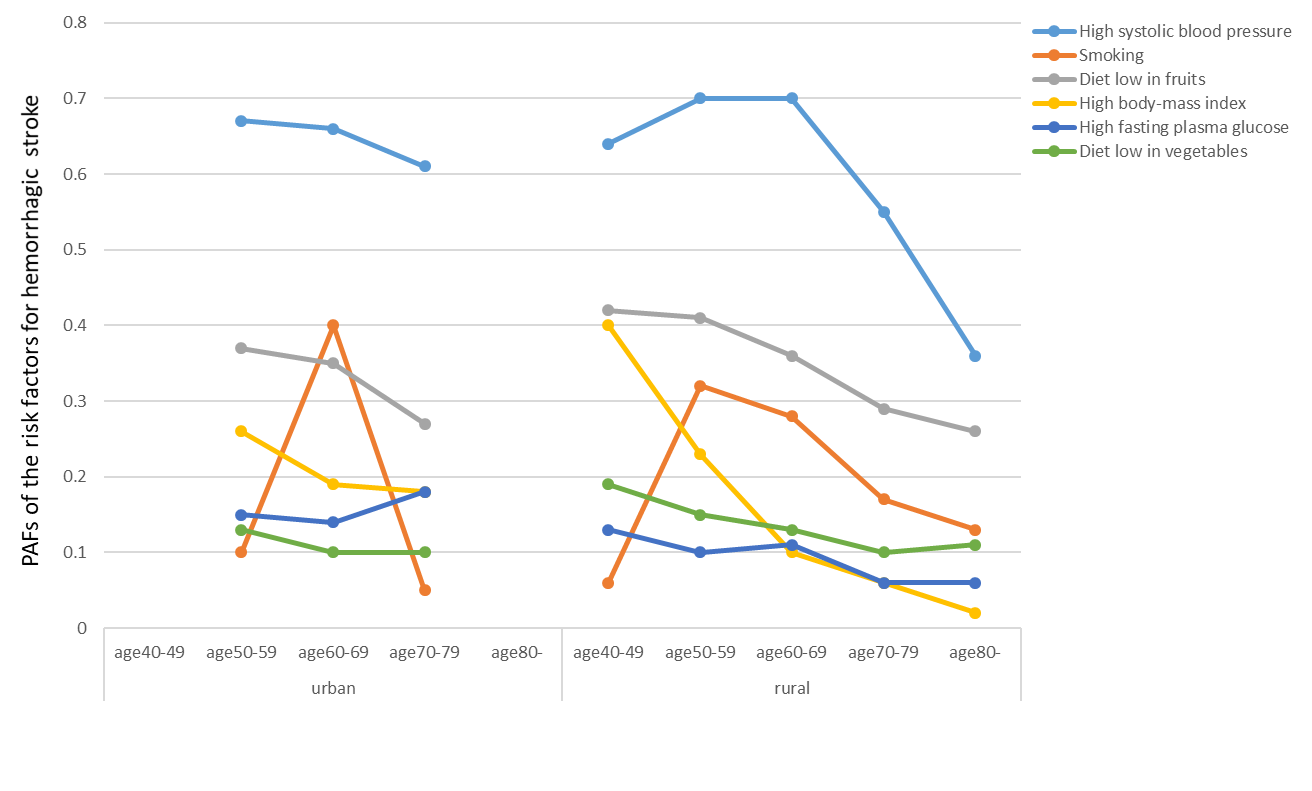
Figure S5. The PAFs of the risk factors for hemorrhagic stroke in urban and rural areas of Sichuan province in 2012 to 2013.
